# A rare case of Allgrove Syndrome associated with growth hormone deficiency in an 8-year-Old child: A case report

**DOI:** 10.1016/j.amsu.2022.104352

**Published:** 2022-08-18

**Authors:** Mahfoud Eid, Ahmad Chreitah, Omar Aljanati, Aria Mohammed, Ibrahim Melhem, Zeina Alkilany

**Affiliations:** a Department of Endocrinology Medicine, Tishreen University Hospital, Latakia, Syria; b Faculty of Human Medicine, Tishreen University, Latakia, Syria

**Keywords:** Triple A syndrome, Achalasia, Alacrimia, Adrenal insufficiency, Growth hormone deficiency

## Abstract

**Introduction and importance:**

Triple A (Allgrove) syndrome is an autosomal recessive multi-organ disease, caused by a mutated gene on chromosome 12q13 in most cases, characterized by the classic triad of Alacrimia, Achalasia and Adrenal insufficiency; along with neurologic abnormalities and many other manifestations in some cases.

While short stature is not a rare manifestation in the context of this syndrome, it remains without identifiable cause.

**Case presentation:**

Here we described an 8-year-old female who had feeding difficulties, recurrent vomiting, hyperpigmentation and short stature. She was diagnosed with Allgrove syndrome after confirmation of adrenal insufficiency, Achalasia, and Alacrimia. Despite correcting these disorders, we did not notice an improvement in the patient's height, which promote us to further investigations, which eventually led to the diagnosis of growth hormone deficiency as a cause of short stature.

The treatment consisted of Hydrocortisone, artificial tears, pneumatic balloon dilation, Nifedipine and Recombinant growth Hormone with a great improvement of her condition.

**Conclusion:**

This case found an unusual association between Allgrove syndrome and growth hormone deficiency, a treatable cause of short stature, which in turn is a frequent manifestation of unknown etiology in this syndrome.

## Highlights

• Allgrove syndrome is a rare multiorgan disorder with a wide range phenotypes.
• Adrenal insufficiency is is the main cause of morbidity and mortality in patient with Allgrove syndrome.
• Short stature may be either a variant of normal growth or caused by a disease.
• Growth hormone deficiency may be isolated or combined with other pituitary hormones deficiency.

## 1 Introduction

Triple A Syndrome (TAS) or Allgrove Syndrome (AS) is a rare multi-organ disorder, transmitted in an autosomal recessive trait, presenting with three main features (Alacrimia, Adrenal insufficiency and Achalasia) [1].

Two-third of patients demonstrate the complete triad and one-third of whom has autonomic nervous system dysfunction [2]. There are many other manifestations described in the literature which include short stature, microcephaly, dysmorphic features and dermatological manifestations [3].

Some authors have reported an estimated prevalence (1 per 1,000,000 individuals) [4], while others have stated that the exact prevalence is unknown and limited to case reports.

This syndrome is caused by a mutated gene located on the chromosome 12q13, which encodes for a protein called ALADIN (Alacrimia–Achalasia–Adrenal Insufficiency–Neurologic disorder) [5]. This protein is mainly expressed in the brain, pituitary gland, cerebellum, adrenal glands, and gastrointestinal tract, which are the organs most affected by this syndrome [2].

Growth hormone deficiency (GHD) is a condition caused by decreased production of growth hormone by the pituitary gland; which could be congenital or acquired, isolated or combined with other pituitary hormone deficiency. The most prominent feature of GHD in children is short stature with an incidence about 1:4000 to 1:10,000 [6].

The main purpose of the case described here is to report a case of AS, which presents with its classic triad as well as short stature caused by growth hormone deficiency. This report is the first to describe this rare association between AS and GHD.

This case report has been reported in line with the SCARE criteria 2020 [7].

### 1.1 Presentation of case

An 8-year-old female issued from a low socio-economic status family was referred to our Pediatric Endocrinology clinic, for evaluation of recurrent vomiting, abdominal pain and short stature.

She was the offspring of consanguineous parents, born at term after a normal pregnancy. Her past medical history revealed three antecedent hospitalizations in the context of convulsions and hypoglycemia, which led to the diagnosis of adrenal insufficiency at the age of 2 years, and she was treated with Hydrocortisone for three years. Her parents also mentioned lack of tears while crying since infancy without any interference. Upon collection of family history, there is a history of the patient's sibling dying for an unknown reason at the age of 3 months.

At admission, the patient was conscious and there were no dysmorphic features. On physical examination, she had hyperpigmentation on various areas of her body rising concern of adrenal insufficiency, with normal detailed neurological assessment.

Her initial vital signs were: pulse: 80 beats/min, respiratory rate: 20 breaths/min, blood pressure: 105/70 mm Hg with no postural drop, Spo 2: 97% of ambient air. Her height was 117 cm and weight was 24 kg at the 3rd and 38th percentiles on CDC growth charts, respectively. The rest of her physical examination was unremarkable.

Laboratory workups revealed a decreased morning cortisol level, with elevated adrenocorticotropic hormone confirming primary adrenal insufficiency. Serum sodium was 138 mmol/L, potassium was 3 mmol/L and the Aldosterone was normal at 4 ng/dl (Table 1), which reflect no Mineralocorticoid deficiency.

**Table 1:** The initial workup of the patient upon admission.

Test	Result	Normal values	Test	Result	Normal values
**WBC**	8 × 10^3^	4.5–11 × 10^3^/μl	CRP	1	Less than 3 mg/dl
**Neutrophil**	60	50–70%	Creatinine	0,6	Less than 1.4 mg/dl
**Lymphocytes**	30	20–40%	BUN	17	7–35 mg/dl
**Hemoglobin**	12.5	More than 11 g/dl	Cortisol	0,28**↓**	5-25 μg/dl
**Platelets**	250 × 10^3^	150–450 × 10^3^/μl	ACTH	827**↑**	7,4–64,3 pg/ml
**Sodium**	138	130–149 mmol/L	Aldosterone	4	2–9 ng/dl
**Potassium**	3	3–5 mmol/L			

^**WBC:**^^Wight blood cell,^^**BUN:**^^Blood urea nitrogen,^^**ACTH**: adrenocorticotropic hormone,^^**CRP:**^^C−reactive protein.^

Ophthalmological consultation revealed Keratopathy and corneal ulceration. Schirmer's test was positive (wetting at 5 min was 0 mm in both eyes) suggestive of Alacrima.

**Fig. 1:**
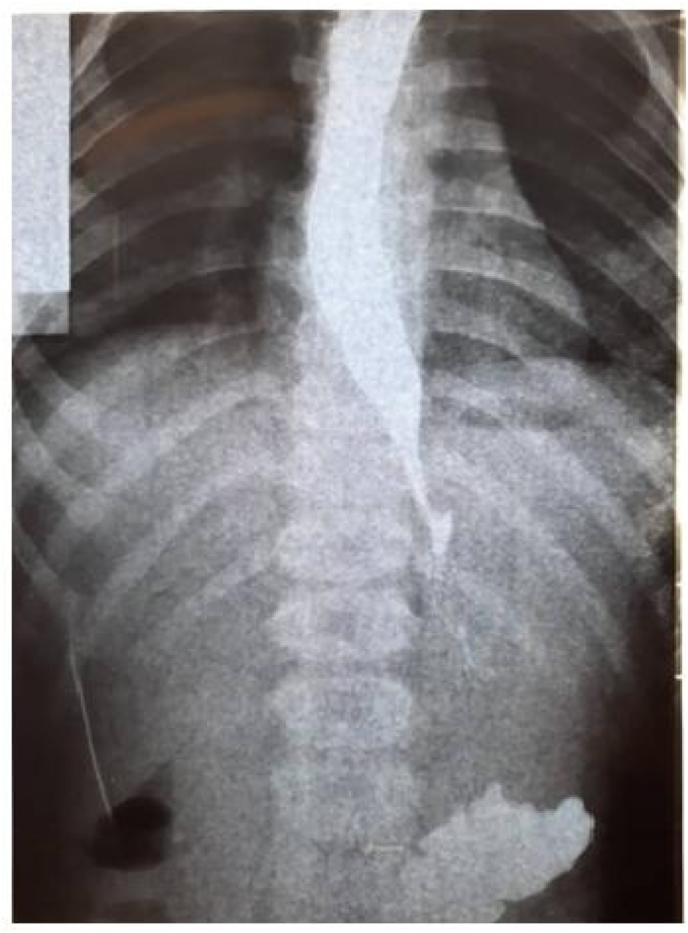
Barium swallow showed “Bird beak sign” suggestive of achalasia.

**Fig. 2:**
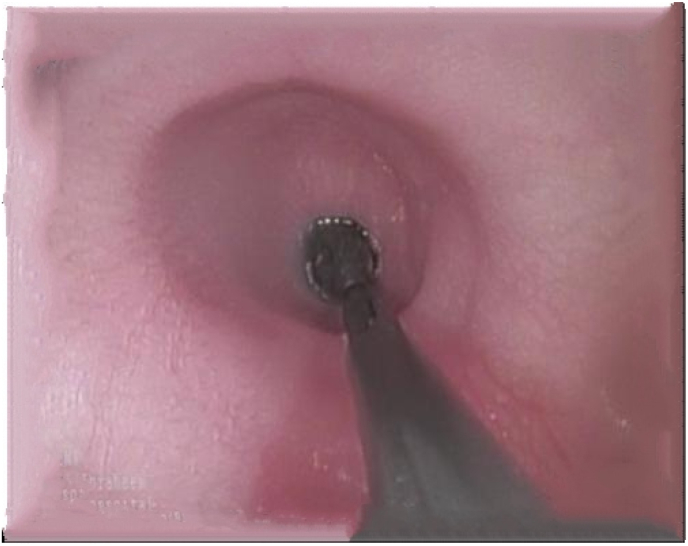
Upper gastrointestinal endoscopy with balloon dilation.

Due to the complaint of abdominal pain with recurrent vomiting, an abdominal ultrasound was performed which was normal. The barium swallow showed “Bird Beak Sign” (Fig. 1), suggestive of achalasia. She underwent an upper endoscopy, which revealed Stenosis of the gastro-esophageal junction with dilation of the upper esophagus, for which she had a pneumatic balloon dilation (Fig. 2). The biopsies showed chronic peptic Duodenitis and chronic active gastritis with marked activity associated with HP infection (+++); for which she was treated with clarithromycin (7.5 mg/kg twice daily), metronidazole (10 mg/kg thrice daily), and Omeprazole (1 mg/kg once daily) for 2 weeks.

Based on the association of adrenal insufficiency, alacrimia and achalasia, a clinical diagnosis of AS was made. We could not establish a genetic diagnosis because it was not available.

Hydrocortisone replacement therapy with (10 mg/m^2^/day) was initiated for adrenal insufficiency and artificial tears for Alacrimia.

Three months later, she had some improvement of abdominal pain, with persistent dysphagia. She underwent a second upper endoscopy, which showed, lower-esophagitis grade A by Los Angeles Classification and the gastro-esophageal junction was normal. The biopsies showed gastric and duodenal mucosa within normal limits as well as negative for HP. She was treated with Sublingual Nifedipine (10 mg/kg once daily) administered 30–45 minutes before meals.

**Fig. 3:**
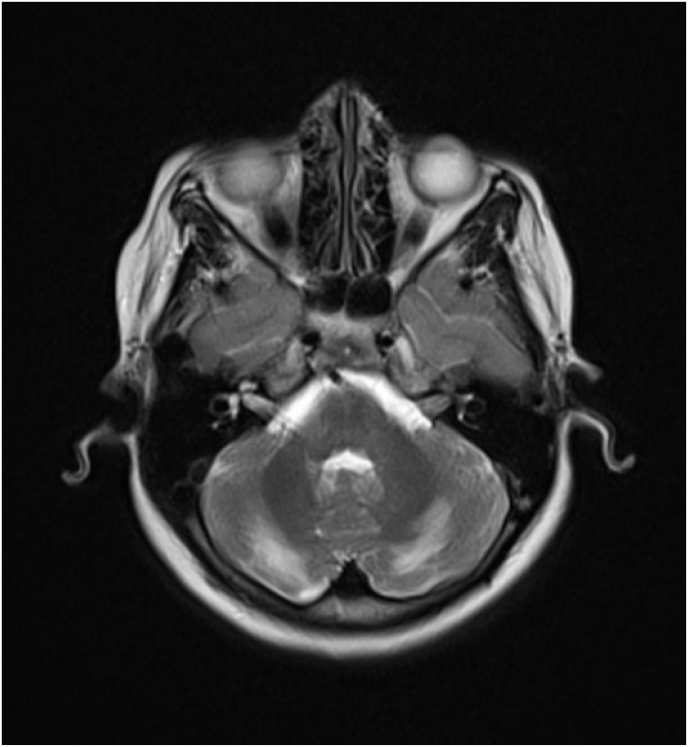
T2-weighted MRI showing high-signal subcortical band in the cerebellar hemispheres.

At the age of 9,5 years, her weight was 28 kg at the 50th percentile, her height was 121 cm below the 5th percentile. After correcting adrenal insufficiency, excluding other causes of growth failure (eg, chronic systemic disease, hypothyroidism, undernutrition) and the patient was growing less than 4.5 mm/yr with delayed bone age (bone age of 8 years), all of this rising concern of GHD, which eventually confirmed by IGF-1 measurement and growth hormone provocation test (Table 2). The magnetic resonance imaging (MRI) of the brain revealed normal pituitary gland with a high-signal Subcortical band in the cerebellar hemispheres, which may be consistent with malacia or dystrophy (Fig. 3). The treatment with subcutaneous recombinant GH was started with an initial dose of 0.24 mg/kg/week, once daily injections.

**Table 2:** Hormonal workup on 18-month follow-up.

Test	Result	Normal values	Test	Result	Normal values
**TSH**	3,5	0.5–5.0 mIU/L	GH	3,9	0,06-5 mIU/L
**FT4**	1,12	0.7–1.9 ng/dL	GH At rest	6,6**↓**	More than 7 ng/ml
**ACTH**	120	10–60 pg/ml	GH after ½ hr	3,7**↓**	More than 7 ng/ml
**Cortisol**	8	5-25 μg/dl	GH after 1 hr	3,2**↓**	More than 7 ng/ml
**ESR**	8	Less than 20 mm/hr	GH after 1,5 hr	3,9**↓**	More than 7 ng/ml
**IGF-1**	>25**↓**	57–316 ng/ml			

^**TSH:**^^thyroid stimulating hormone,^^**FT4:**^^free thyroxine,^^**ACTH**: adrenocorticotropic hormone,^^**IGF−1:**^^insulin-like growth factor 1,^^**GH:**^^growth hormone,^^**ESR**: erythrocyte sedimentation rate.^

## 2 Discussion

This present case described a young child, diagnosed with Allgrove syndrome based on the presence of its cardinal features (Alacrimia, Achalasia, Adrenal insufficiency), a condition which is very rare, with approximately 200 cases reported since the first description in 1978 by Allgrove.

Short stature and poor growth are not uncommon features in AS. Several reports such as Duseck T et al. [8] described this association, but the exact mechanism has not been established; in addition, one case report revealed a coincidental association between Laron syndrome and AS [9]. The short stature and poor growth in this syndrome may be due in part to adrenal insufficiency or nutritional difficulties due to achalasia. However, in our case GHD was the cause of short stature, and to the best of our knowledge this unusual association between AS and GHD, has not been reported to date.

Achalasia is defined as failure of the lower esophageal sphincter (LES) to relax, accompanied by a loss of peristalsis in the distal esophagus. Alhussaini B et al., demonstrated that AS patients frequently showed high lower esophageal sphincter pressures on Manometry compared with other Achalasia patients, which was associated with treatment failure [10].

Although most cases begin in the first decade of life, the age of onset of symptoms varies greatly, with Alacrimia being the most constant symptom of this syndrome with approximately 90% of cases [3]. However, because it often goes unnoticed or overlooked, this makes dysphagia, frequent vomiting and episodes of hypoglycemia the most presenting symptoms of the disease.

Primary adrenal insufficiency in AS is due to unresponsiveness of the adrenal gland to ACTH and it is the main cause of morbidity and mortality in these patients. Glucocorticoid function is lacking in most cases, while Mineralocorticoid deficiency is reported only in a minority of patients (15%) [4], so that its clinical presentation may be similar to disorders of central adrenal insufficiency.

AS may associated with neurologic disorders affecting the central, peripheral and autonomic nervous system. Clinical manifestations depend on the affected system. In our case there are no neurologic abnormalities, yet and the findings on MRI may be due to reduced cerebellum Purkinje cells which are likely to be seen in AS patients as demonstrated by Giacomo Bitetto et al. [11]. These findings may indicate that our patient may subsequently develop ipsilateral limb Dysmetria, hypotonia, tremor, and ataxia if the involvement is exacerbated or extended to the Vermis; so that lifelong monitoring is needed as there is potential for developing other new manifestations, even decades after the initial diagnosis, as well the potential need for other interventions such as Heller myotomy in cases where cardias Stenosis persists.

We assure that Allgrove syndrome is a multisystem disorder with a wide range of phenotypes, which can affect any age, so it should be kept in mind in everyone has any of its essential features in order to make an early diagnosis and avoid potentially harmful complications.

## 3 Conclusion

Although AS is described and its main clinical manifestations are well known, it can be associated with other manifestations, some of which are still unexplained such as short stature and growth failure, so we suggest to consider growth charts, bone age, IGF-1 and MRI for the brain and pituitary gland in each case of short stature in the context of this rare syndrome due to the possibility of a treatable cause.

## Ethical approval

This case report did not require review by the Ethics Committee Tishreen university hospital, Latakia, Syria.

## Sources of funding

This research did not receive any specific grant from funding agencies in the public, commercial, or not-for-profit sectors.

## Author contribution

Mahfoud Eid: contributed in study concept and design, data collection.

Ahmad Chreitah: contributed in data interpretation.

Omar Aljanati: contributed in writing the paper.

Aria Mouhammad:contributed in writing the paper.

Ibrahim Melhem: contributed in writing the paper.

Zeina alkilany: contributed in writing the paper.

## Registration of research studies

Not applicable.

## Guarantor

Mahfoud Eid.

## Consent

Written informed consent was obtained from the patient's parents for publication of this case report and accompanying images. A copy of the written consent is available for review by the Editor-in-Chief of this journal on request.

## Provenance and peer review

Not commissioned, externally peer reviewed.

## Declaration of competing interest

All of the authors declare that they have no competing interests.

## References

[1] Allgrove J., Clayden G.S., Grant D.B., Macaulay J.C. (1978). Familial glucocorticoid deficiency with achalasia of the cardia and deficient tear production. Lancet.

[2] Huebner A., Yoon S.J., Ozkinay F. (2000). Triple A syndrome–clinical aspects and molecular genetics. Endocr. Res..

[3] Huebner A., Elias L.L., Clark A.J. (1999). ACTH resistance syndromes. J. Pediatr. Endocrinol. Metab..

[4] Flokas (2019). Triple A syndrome (Allgrove syndrome): improving outcomes with a multidisciplinary approach. Pediatr. Health Med. Therapeut..

[5] Weber A., Wienker T.F., Jung M., Easton D., Dean H.J., Heinrichs C. (1996). Linkage of the gene for the triple A syndrome to chromosome 12q13 near the type II keratin gene cluster. Hum. Mol. Genet..

[6] Rona R.J., Tanner J.M. (1977). Aetiology of idiopathic growth hormone deficiency in England and Wales. Arch. Dis. Child..

[7] Agha R.A., Franchi T., Sohrabi C., Mathew G., for the SCARE Group (2020). The SCARE 2020 guideline: updating consensus surgical CAse REport (SCARE) guidelines. Int. J. Surg..

[8] Dusek T., Korsic M., Koehler K., Perkovic Z., Huebner A., Korsic M. (2006). A novel AAAS gene mutation (p.R194X) in a patient with triple A syndrome. Horm. Res..

[9] (2012). Triple A syndrome in a patient with genetic growth hormone insensitivity: phenotypic effects of two genetic disorders silvia. Marin Horm Res Paediatr.

[10] Alhussaini B., Gottrand F., Goutet J.M. (2011). Clinical and manometric characteristics of Allgrove syndrome. J. Pediatr. Gastroenterol. Nutr..

[11] Bitetto Giacomo (2019 Dec 1). Loss of the nucleoporin Aladin in central nervous system and fibroblasts of Allgrove Syndrome. Hum. Mol. Genet..

